# Neural Correlates of Restless Legs Syndrome (RLS) Based on Electroencephalogram (EEG)—A Mechanistic Review

**DOI:** 10.3390/ijms262110675

**Published:** 2025-11-02

**Authors:** James Chmiel, Donata Kurpas

**Affiliations:** 1 Institute of Neurofeedback and tDCS Poland, Ul. 3 Maja 25–27, 70-215 Szczecin, Poland; 2 Department of Family and Pediatric Nursing, Faculty of Health Sciences, Wrocław Medical University, 51-618 Wroclaw, Poland; donata.kurpas@umw.edu.pl

**Keywords:** restless leg syndrome, RLS, electroencephalography, electroencephalogram, EEG, QEEG, neurophysiology, neural correlates, oscillations, ERP

## Abstract

Restless legs syndrome (RLS) is a sensorimotor disorder with evening-predominant symptoms; convergent models implicate brain iron dysregulation and alter dopaminergic/glutamatergic signaling. Because EEG provides millisecond-scale access to cortical dynamics, we synthesized waking EEG/ERP findings in RLS (sleep EEG excluded). A structured search across major databases (1980–July 2025) identified clinical EEG studies meeting prespecified criteria. Across small, mostly mid- to late-adult cohorts, four reproducible signatures emerged: (i) cortical hyperarousal at rest (fronto-central beta elevation with a dissociated vigilance profile); (ii) attentional/working memory ERPs with attenuated and delayed P300 (and reduced frontal P2), pointing to fronto-parietal dysfunction; (iii) network inefficiency (reduced theta/gamma synchrony and lower clustering/longer path length) that scales with symptom burden; and (iv) motor system abnormalities with exaggerated post-movement beta rebound and peri-movement cortical–autonomic co-activation, together with evening-vulnerable early visual processing during cognitive control. Dopamine agonist therapy partially normalizes behavior and ERP amplitudes. These converging EEG features provide candidate biomarkers for disease burden and treatment response and are consistent with models linking brain iron deficiency to thalamo-cortical timing failures. This mechanistic review did not adhere to PRISMA or PICO frameworks and did not include a formal risk-of-bias or quantitative meta-analysis; samples were small, heterogeneous, and English-only.

## 1. Introduction

Restless legs syndrome (RLS), also known as Willis–Ekbom disease, is a common neurological sensorimotor disorder characterized by an irresistible urge to move the legs, usually accompanied by unpleasant sensations in the lower limbs [1]. The defining feature is that symptoms begin or worsen during periods of rest or inactivity (especially in the evening or at night) and are partially or completely relieved by movement such as walking or stretching [2]. Patients often describe diffuse, difficult-to-localize sensations—commonly using terms like crawling, tingling, creeping, or itching deep in the legs—that drive an urge to move in order to obtain relief [3]. The disorder predominantly affects the legs, but in severe cases, symptoms may spread to the arms or other body parts [4]. Many individuals with RLS also experience periodic limb movements of sleep (PLMS), which are involuntary, rhythmic leg jerks during sleep; these occur in up to 80–90% of RLS patients and can contribute to sleep fragmentation, though PLMS are not specific to RLS [5]. Clinically, RLS can be idiopathic (primary) or associated with other conditions such as iron deficiency, pregnancy, end-stage renal disease, or neuropathy [6]. Regardless of cause, RLS symptoms often lead to significant sleep disturbance, daytime fatigue, and reduced quality of life if not adequately treated [7].

RLS is now recognized as a widespread disorder, although prevalence estimates vary with methodology [8]. Extensive population studies in North America report that roughly 10% of adults experience RLS symptoms, with around 2–3% having clinically significant symptoms that are frequent or severe enough to require treatment [9]. Prevalence increases with age through mid-life, and some estimates in older adult populations (elderly over ~60–70 years) have ranged as high as 10–35% [10], though prevalence may decline in extreme old age [11]. Most studies state that it affects women about twice as often as men [12]. Notably, pregnancy is a strong precipitant of RLS symptoms: approximately one-third of women experience RLS in the third trimester [13], and higher parity (number of pregnancies) is associated with increased risk of RLS, which may partially explain the female predominance [14].

Although the exact pathophysiological mechanism of RLS remains incompletely understood, two interconnected factors are central in current models: brain iron deficiency and dopaminergic dysfunction [15]. The link between iron and RLS has long been recognized—RLS symptoms often improve with iron supplementation and worsen with iron loss—yet many patients have normal peripheral iron levels, suggesting that the issue lies in iron regulation within the brain [16]. However, studies using MRI and cerebrospinal fluid (CSF) analysis have demonstrated that RLS patients frequently have increased brain iron content (increased iron signal in the caudate) [17]. Iron is a crucial cofactor for tyrosine hydroxylase, which is the enzyme responsible for dopamine synthesis [18]. Therefore, brain iron insufficiency could impair dopamine production or signaling in critical areas of the central nervous system [19]. Paradoxically, the dopaminergic abnormalities in RLS do not simply mimic a classic dopamine deficiency. At the same time, early brain imaging studies in RLS were somewhat conflicting; a prevailing hypothesis is that iron deficiency leads to altered dopamine neurotransmission characterized by increased dopamine activity in some contexts and times (especially as a compensatory response), coupled with relative dopamine hypoactivity during the night when symptoms arise [2]. This aligns with clinical observations: RLS symptoms typically peak at night when endogenous dopamine levels are lowest, and medications that enhance dopaminergic transmission (levodopa or dopamine agonists) markedly alleviate symptoms [20], whereas antidopaminergic drugs can precipitate or worsen RLS. In addition to iron and dopamine, several other neurochemical systems appear to play a role. Glutamate levels are elevated in the thalamus of RLS patients, suggesting a state of hyperglutamatergic neurotransmission that may contribute to both the sensory symptoms and the sleep disruption [21]. This is supported by the efficacy of gabapentinoids (α2δ calcium channel ligands like gabapentin and pregabalin), which reduce glutamate release and are effective in RLS treatment [22], as well as by the observation that methadone, which has both opioid agonist and NMDA antagonist (anti-glutamate) properties, is particularly effective in severe RLS [23]. The endogenous opioid system itself might be involved in RLS pathophysiology: some RLS patients have reduced CSF opioid levels, and opioids provide relief, implying a possible deficiency of inhibitory opioid tone in RLS [24,25]. Additionally, recent research has implicated adenosine—a sleep-promoting neurotransmitter—in RLS: brain iron deficiency may trigger a downregulation of adenosine A1 receptors (hypoadenosinergic state), which in turn could produce both movement urges and a state of heightened arousal [26].

Emerging endocrine data suggest that thyroid status may modulate RLS biology alongside iron–dopamine pathways. A hospital-based cohort reported higher serum TSH and a markedly increased prevalence of subclinical hypothyroidism in RLS versus controls, with TSH independently associated with RLS risk. At the same time, se-based evidence links both Graves’ disease and Hashimoto’s thyroiditis to RLS onset or worsening—together implying bidirectional interactions between thyroid hormones, dopaminergic tone, and iron metabolism in susceptible patients [27,28,29,30].

At the genetic level, a 2024 genome-wide meta-analysis (116,647 cases; 1.55 M controls) expanded the number of RLS risk loci to 164 (including X-chromosome signals), refining implicated pathways and improving polygenic risk prediction, while epigenome-wide methylation work supports peripheral biomarkers and regulatory mechanisms relevant to iron and dopaminergic signaling [31,32].

Finally, the gut–brain axis may influence RLS via dopaminergic signaling and iron handling. Reviews and early clinical studies point to increased intestinal permeability (e.g., elevated zonulin) and dysbiosis as potential contributors to systemic inflammation, altered iron absorption, and neuromodulation via microbial metabolites (e.g., short-chain fatty acids), offering testable links between microbiota, iron availability, and dopamine-dependent circuits in RLS [33,34,35,36].

Neurobiological evidence shows that RLS is a disorder associated with nervous system dysfunction. Modern medicine and neuroscience have tools that measure brain activity, structure, and connectivity. The oldest, most basic, and routinely used neuroimaging method is electroencephalography (EEG).

The scalp EEG is a continuous record of the brain’s spontaneous electrical activity, generated by the summed postsynaptic currents of large cortical neuron populations and captured at the scalp with millisecond precision. Instead of the discrete peaks that characterize event-related potentials, typical (“resting” or “ongoing”) EEG is described as a mixture of rhythmic bands whose center frequencies and topographies reveal the brain’s moment-to-moment state. Five canonical ranges are recognized: delta (≈0.5–4 Hz), theta (≈4–7 Hz), alpha (≈8–12 Hz), beta (≈13–30 Hz), and gamma (>30 Hz) [37]. In healthy adults, the eyes-closed alpha rhythm, which is the strongest over occipital cortex, is the dominant background activity; it diminishes when the eyes open or attention shifts outward, illustrating how intrinsic oscillations track engagement with the environment [38,39]. Longitudinal and large-sample studies show that the frequency of this alpha peak accelerates from roughly 6 Hz in infancy to just over 10 Hz in the late 20s, then slows again with advancing age. In contrast, overall alpha power gradually declines—age-related trends that make the alpha profile a useful developmental and geriatric biomarker [40,41].

Each band is linked to broad functional themes: delta dominates deep non-REM sleep [42]; theta rises during drowsiness and memory formation [43]; alpha reflects an “idling but ready” visual network [44]; beta increases over sensorimotor cortex during movement or focused thought [45]; and gamma synchrony is implicated in perceptual binding and working memory [46,47]. Deviations from these normative patterns often foreshadow or accompany disease. For example, a shift from posterior alpha toward slower theta–delta activity in quiet wakefulness correlates with amyloid burden and cognitive decline in prodromal Alzheimer’s disease, and automated EEG pipelines are now being tested as low-cost screening tools [48].

Because a typical EEG is non-invasive, inexpensive, and portable, it can be recorded across the lifespan and in bedside settings, offering a real-time readout of cortical excitability and network coordination. Its millisecond temporal resolution captures dynamics invisible to functional MRI, yet the technique is susceptible to ocular and muscular artifacts and suffers from limited spatial precision. Nevertheless, when carefully processed and interpreted, the continuous background EEG provides a general but powerful lens on brain physiology—revealing how intrinsic rhythms evolve with development, adapt to cognitive demands, and diverge in aging and disease.

Event-related potentials, or ERPs, are the tiny voltage deflections in the scalp EEG when many repetitions of the same event are averaged together. This process strips away the larger background rhythms and leaves only the millisecond-precise electrical response typical to every trial [49]. These signals arise not from isolated action potentials but from the summed postsynaptic currents of vast, synchronously active assemblies—about ten million—of cortical pyramidal neurons whose dendrites point perpendicularly toward the skull, allowing their fields to add up instead of canceling out. This is because the scalp pattern predicted for a known generator can be calculated. At the same time, the reverse inference cannot; ERPs deliver exquisite temporal precision at the price of uncertain spatial localization, which is a challenge encapsulated in the classic forward-versus-inverse problem.

When plotted (by convention with negative upward), a stimulus-locked waveform reveals an orderly cascade: early sensory components such as C1, P1, and N1; the mid-latency N2 that flags novelty or conflict; and the broad parietal P300 that indexes context updating and working memory operations. Oddball paradigms, in which rare deviant stimuli punctuate a stream of standards, reliably provoke the N200–P300 complex and have become a staple for dissecting selective attention and stimulus evaluation [50].

Because every peak and trough is timestamped in real time, ERPs offer a millisecond-by-millisecond chronometry of perception, cognition, and action that even the fastest imaging techniques cannot match [49]. Their non-invasive and inexpensive nature makes them suitable across the lifespan and in clinical settings. Characterization alterations in components such as the P300 or mismatch negativity have been tied to disorders ranging from schizophrenia and alcoholism to Parkinson’s disease and dementia, underscoring their growing translational value [50]. Yet the features that make ERPs attractive also impose limits: amplitudes of only a few microvolts demand hundreds of artifact-free trials; eye blinks, muscle tension, and electrical noise can swamp the signal; and the inverse problem forever tempers anatomical certainty [49]. Even so, ERPs remain one of the most transparent windows into the brain’s fast electrical language when meticulously recorded and interpreted.

This mechanistic review aims to identify and synthesize all EEG studies to measure brain bioelectrical activity in patients with RLS. We sought to answer whether EEG parameters in individuals with RLS differ from those of healthy controls and whether common neurophysiological correlates of this disorder can be found across studies that may explain the occurrence of cognitive–behavioral changes. Furthermore, the most essential part of the paper is a discussion of the potential pathophysiological mechanisms of RLS based on EEG. EEG measurements during sleep are not included in this review, as this is an area of interest in sleep medicine and has already been discussed in the literature. Moreover, we excluded sleep EEG studies to avoid state-dependent and methodological confounds that would blur cortical inferences. Sleep microstructure (notably the cyclic alternating pattern, CAP) and frequent arousals make the sleeping brain an intrinsically unstable target; CAP varies across NREM stages and disease states and can dominate spectral findings independently of any disorder-specific cortical mechanism. Harmonizing results across papers that quantify CAP/arousals differently is therefore difficult. Technically, sleep EEG around limb movements is highly susceptible to movement and EMG artifacts and lead instabilities, which inflate or distort power estimates—problems persist even with modern detection pipelines.

## 2. Methods

This mechanistic review aims to collect and synthesize EEG findings in restless leg syndrome. Although it draws on some principles of systematic reviews—such as structured search and study selection—its primary goal is to explain the underlying brain correlates identified through EEG in restless leg syndrome. Given its mechanistic focus, this review does not follow the full PRISMA protocol typically required for systematic reviews. It also omits formal assessments like risk of bias or the PICOS framework.

### 2.1. Data Sources and Search Strategy

To identify relevant studies, two researchers (J.Ch. and D.K.) independently conducted a comprehensive literature search using a combination of keywords related to EEG and restless leg syndrome. The search terms included “EEG”, “QEEG”, “electroencephalogram”, “electrophysiology”, “neurophysiology”, “event-related potential”, “ERP”, “ERPs”, paired with “restless leg syndrome” and “restless leg”, among others. The search was carried out in July 2025 and included multiple databases—PubMed/Medline, Scopus, ResearchGate, Google Scholar, and Cochrane—focusing on studies published from January 1980 to July 2025. In addition, the Google browser was also used, and similar and cited articles were searched in the PubMed database. Bibliographies of relevant literature were also checked to identify studies within the scope of this review.

### 2.2. Study Selection Criteria

Publications must be case studies or clinical trials published in English between January 1980 and July 2025 to be eligible for inclusion. Papers written in languages other than English were not included.

### 2.3. Screening Process

A multi-step screening process was used to select studies that met the inclusion criteria. First, the titles and abstracts of all retrieved studies were reviewed independently by both researchers for relevance.

#### 2.3.1. Title and Abstract Screening

Each reviewer independently evaluated the titles and abstracts to identify papers that discussed EEG use in restless leg syndrome patients. Only those aligned with the topic were considered for the next stage.

#### 2.3.2. Full-Text Assessment

Articles that passed the initial screening were then assessed in full to confirm that they met all eligibility criteria, particularly that they were clinical trials involving restless leg syndrome patients and EEG assessment, which were published in English between January 1980 and July 2025.

## 3. Results

**Figure 1:**
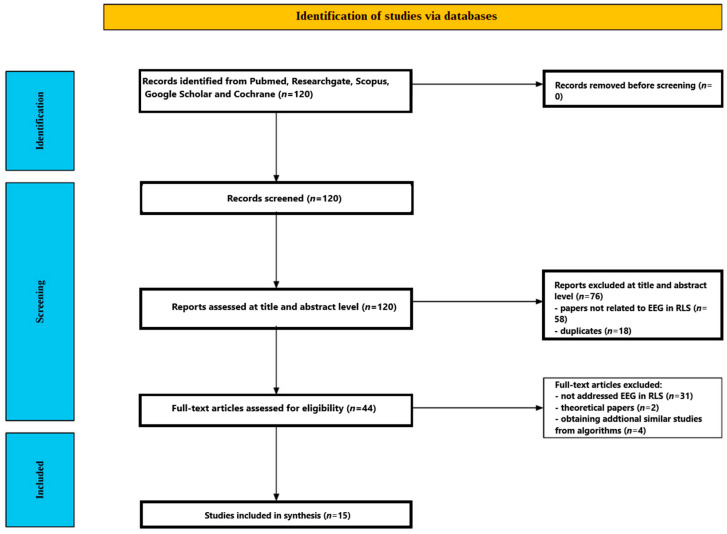
Flow chart depicting the different phases of the systematic review.

Figure 1 shows the flow of the screening process. The initial database search yielded 120 studies. After reviewing the titles and abstracts, 76 papers were excluded—58 for being unrelated to EEG in restless leg syndrome, and 18 as duplicates. The remaining forty-four articles underwent full-text review, during which thirty-one were excluded for not directly addressing EEG in restless leg syndrome, and two articles were excluded because they did not record EEG but proposed a new theory based on EEG results. An additional four articles were found through PubMed algorithms proposing similar studies to those matching the criteria of this review. In total, 15 studies met all inclusion criteria and were selected for the final analysis [51,52,53,54,55,56,57,58,59,60,61,62,63,64,65]. Studies included in the review are presented in Table 1.

### 3.1. Participants

Across the 15 EEG investigations, 657 individuals were studied: 402 patients with idiopathic RLS and 255 comparison participants (healthy controls = 212; primary insomnia = 17; periodic-limb-movement disorder = 26; some studies contributed to more than one comparison group).

#### 3.1.1. Sample Size and Sex Distribution

Most experiments were small and laboratory-based, enrolling between 10 and 33 patients apiece; only the quantitative connectivity study recruited a larger retrospective cohort (n = 107 RLS). Except where explicitly mixed, the samples were predominantly female (six studies used all-female RLS cohorts). Male representation, therefore, ranged from none to 45% and averaged ~27% across the full dataset.

#### 3.1.2. Age

All studies concerned mid- to late-adulthood. Reported mean ages clustered narrowly around 50–55 years, with the youngest cohort averaging 45.5 years and the oldest 65.2 years. No pediatric or very-old (>75 y) samples were included.

#### 3.1.3. Diagnostic Confirmation and Disease Severity

Every study applied contemporary International RLS Study Group criteria, typically supplemented by a structured clinical interview and a validated severity scale (most often the International RLS Severity Scale, with mean baseline scores 19–30, indicating moderate-to-severe disease). Three investigations [51,52,55] were additionally screened with the Korean or English versions of the Johns Hopkins RLS diagnostic questionnaire.

#### 3.1.4. Medication Status and Comorbidity

Eleven of the thirteen datasets studied drug-naïve or drug-free patients; the remaining two examined (i) pramipexole-naïve patients before and after 12–16 weeks of therapy and (ii) chronically affected out-patients undergoing routine care but off psychotropics for ≥2 weeks. All protocols excluded secondary RLS (e.g., renal failure, iron-deficiency anemia), significant psychiatric or neurological comorbidity (Parkinson’s disease, ADHD, peripheral neuropathy), and, where sleep was a confound, untreated sleep apnea.

#### 3.1.5. Control and Comparison Groups

Ten studies matched healthy controls one-to-one on age (±3 y) and sex; two used both healthy controls and a clinical comparator group (periodic-limb-movement disorder or primary insomnia); one employed only primary-insomnia comparators. All healthy controls underwent either overnight polysomnography or a structured sleep interview to confirm the absence of sleep disorders.

#### 3.1.6. Screening Instruments

Nearly every protocol incorporated standard psychometric and sleep questionnaires—Pittsburgh Sleep Quality Index, Epworth Sleepiness Scale, Insomnia Severity Index, Beck Depression and Anxiety Inventories—to characterize mood, daytime vigilance, and sleep quality; scores in the RLS arms consistently indicated poorer sleep and higher depressive symptomatology than in control arms yet remained below diagnostic thresholds for major depression or hypersomnia.

### 3.2. EEG Paradigms

EEG methods across the included studies spanned task-evoked ERPs, resting/quantitative EEG (QEEG) spectral mapping, movement- or state-provoked activation protocols, and network/connectivity analyses. Most studies used standard 10–20 scalp montages (19–27 channels; range 19–27, with a few 21–23 channel variants) and morning recordings to minimize circadian symptom load. However, several paradigms explicitly manipulated time of day or motor state. Below, we group paradigms by experimental class and highlight the cognitive or physiological construct each was designed to probe.

#### 3.2.1. Working Memory (Sternberg) Paradigms

Four studies implemented Sternberg-type digit memory sets with probe recognition to test short-term maintenance and retrieval [51,53,55,57]. Memory load was varied (2–4 items), and responses were speeded yes/no decisions to a probe item presented after a brief retention interval. EEG was time-locked to probe onset to index retrieval/decision processes.

(a) Primary ERP focus: P300 (parietal and frontal amplitudes; latency secondary) in [51] and treatment response extension [53]; theta-band power dynamics and interregional phase synchrony during retrieval in [55]; and single-trial ERP topographies (150–250 ms) entered into explainable deep-learning classifiers in [57].
(b) Acquisition details: 19 channels with 10–20 recordings in [51,53,55,57]; standard preprocessing (filtering, artifact rejection/ICA, epoching; baseline windows per study).
(c) Key analytic additions: Graph-theoretic small-world metrics derived from theta synchrony [55]; sLORETA current-density mapping and layer-wise relevance propagation to identify cortical contributors to group separability [57].

#### 3.2.2. Visual Oddball Attention Paradigms

Three closely related reports examined unmedicated female RLS cohorts performing a visual oddball target-detection task [42,54,56]. Participants responded to infrequent shapes (targets) embedded among frequent standards.

(a) ERP components: Classic P300 (amplitude/latency) in [52]; extended component set including P2 (150–250 ms) and P3 (300–450 ms) with source localisation (LORETA) to medial prefrontal/anterior cingulate and precuneus generators in [54]; combined ERP + induced/evoked gamma-band activity and gamma phase synchrony (GBPS) with network metrics in [56].
(b) Recording: 27-channel montage in [52]; multichannel (reported as “frontal-central emphasis”) arrays in [54,56] (same core cohort as [52]). Alternating eyes-open/closed resting runs were also acquired pre-task in [52] to assess vigilance and derive broadband relative power (delta → beta2).

#### 3.2.3. Circadian Cognitive-Control (Flanker) Paradigm

Study [49] assessed dopaminergically sensitive attentional control by administering a standard Eriksen flanker task in morning (08:00–09:00) vs. evening (17:00–18:00) sessions. EEG captured stimulus-locked ERPs; analyses targeted visual sensory-attentional components (P1, N1) and later conflict/decision components (N2, P3). sLORETA source modeling linked circadian N1 modulation to extrastriate visual cortex (BA 18).

#### 3.2.4. Vigilance-Controlled and Resting-State QEEG Mapping

Broadband spectral characterization under relaxed wakefulness was central to several studies:

(a) Vigilance-controlled morning EEG (V-EEG): 3 min eyes-closed recordings after two lab nights in drug-free RLS vs. controls [60]; 21-channel montage; 36 derived spectral variables spanning 1.3–35 Hz bands (delta → beta-5) plus centroids/dominant frequency indices, extensive omnibus statistics, and topographic probability mapping.
(b) Daytime mapping with multimodal cohort (RLS, PLMD, controls): Parallel 1.3–35 Hz spectral quantification and psychometric correlation in [61]; subset with overnight PSG.
(c) Eyes-closed resting QEEG connectivity (RLS vs. primary insomnia): Five-minute recordings; source-space ciPLV connectivity across canonical bands (delta → gamma) controlling demographic and affective covariates in a large retrospective sample [58].
(d) Alertness-check resting blocks embedded in oddball design: Alternating 20 s eyes-closed/open epochs repeated six times in [52] to ensure wakefulness and to compute relative band powers.
(e) Resting-state EEG with standard activation procedures such as photic stimulation and hyperventilation [64].

#### 3.2.5. Motor-State/Movement-Provocation Paradigms

(a) Suggested Immobilization Test (SIT): One-hour quiet-wake limb-rest period before sleep in 53 drug-free patients; continuous EEG + heart rate capture around spontaneous leg movements categorized as periodic (PLM), isolated (ILM), or short-interval (SILM); 40 s peri-movement windows (−20/+20 s) analyzed for delta → beta absolute power trajectories and autonomic coupling [62].
(b) Simple Paced Motor Response: Right-index button presses to auditory clicks (~6 s ISI) to elicit sensorimotor beta event-related desynchronization/synchronization (ERD/ERS) over C3/Cz; two beta sub-bands (14–20; 20–32 Hz) quantified for movement-related reactivity and post-movement rebound [63].
(c) SIT: EEG data were processed offline to analyze movement-related changes in mu and beta rhythms. Specifically, the study examined ERD and post-movement beta synchronization (PMBS) [65].

#### 3.2.6. Derived Network/Oscillatory Metrics Across Paradigms

Several investigations layered connectivity or network topology analyses atop traditional ERP/spectral measures: theta-weighted phase-lag index (wPLI) and graph metrics (clustering, path length, small-world propensity) during WM retrieval [55]; gamma-band phase synchrony (GBPS) and graph topology aligned to the P300 window in oddball performance [56]; ciPLV source-space connectivity differentiating RLS from insomnia under rest [58]; and relevance-mapped cortical networks from CNN-classified single-trial ERPs in modified Sternberg WM [47]. Collectively, these approaches converge on fronto-parietal integrative dysfunction as a recurring system-level feature of RLS.

### 3.3. EEG Measures and Outcomes

Electroencephalography has provided converging evidence that reproducible alterations in spontaneous and task-related brain activity accompany the cognitive—affective burden of RLS. The findings span conventional spectral analyses, ERPs, oscillatory synchrony, graph theoretical metrics, and treatment-induced plasticity. Together, they outline a profile of cortical hyperarousal, impaired fronto-parietal processing, and network inefficiency that parallels the behavioral phenotype of slowed but generally accurate performance.

#### 3.3.1. Resting-State Spectral Signatures

The study’s [52] analysis of the resting-state EEG data revealed that RLS patients had significantly increased beta band activity (~26–30 Hz) in frontal and central brain regions, suggesting heightened cortical arousal or activation. Though standard ANOVA tests did not detect between-group differences across all frequency bands, more sensitive cluster-based nonparametric statistical methods confirmed the increase in beta activity among RLS patients.

In the study [61], the EEG profile bore a striking resemblance to that typically seen in individuals with major depression. Specifically, there was a significant increase in delta and fast alpha power and a decrease in slow alpha power. Furthermore, the centroid frequency of the delta/theta band was slowed, whereas the centroid of alpha and the dominant frequency were both accelerated. Despite this acceleration, the absolute power of the dominant frequency was attenuated. Interestingly, total EEG power did not differ significantly from controls.

When examining EEG results, the study [64] found that 56.5% of RLS patients exhibited background beta activity, characterized by 14–20 Hz frequency and 5–10 µV amplitude. Additionally, 34.8% had mild paroxysmal slow-wave activity superimposed on an alpha rhythm, and 8.7% showed a pure alpha rhythm. In contrast, the control group exhibited 75% alpha rhythm, 20% beta activity, and only 5% had mild paroxysmal patterns. Statistical comparison showed a significant difference between the two groups, indicating that EEG beta activity was more prevalent among RLS patients. Further analysis revealed a strong association between insomnia and beta activity in the EEG. Of the RLS patients who had insomnia, 57.9% displayed beta activity, and the majority of these were women. Interestingly, 84.6% of all patients with beta EEG patterns also reported insomnia. Even among those with a complete therapeutic response to medication, more than half continued to suffer from insomnia, and most of these had beta activity on the EEG.

#### 3.3.2. Vigilance Markers

In the study [60], participants underwent two nights of polysomnographic recording in a sleep lab, followed by a vigilance-controlled EEG (V-EEG) recording the next morning (3 min of recording). Repeated-measures MANOVA confirmed significant differences across five key frequency bands (delta, theta, alpha-1, alpha-2, and beta), especially in anterior brain regions, notably both frontopolar, right frontal, frontotemporal, central, parietal, and left temporal areas. Univariate analyses provided more granularity. RLS patients exhibited a significant increase in absolute delta and fast alpha (alpha-2) power, particularly over the left occipitotemporal region. Conversely, slow alpha (alpha-1) power was significantly reduced over parietal areas and showed a trend towards reduction in frontal and right frontotemporal regions.

Additionally, there was a relative increase in alpha-2 power across the cortex, most prominently in occipital, left occipitotemporal, and right frontal areas. Relative beta-3 power was also elevated in the right parietal region. Absolute beta-5 power was decreased right frontally. Total power was attenuated over the left occipitotemporal region. Spectral centroid analyses showed a slowing of the delta/theta centroid (indicative of increased low-frequency activity) and an acceleration of the alpha centroid and dominant frequency, pointing toward altered vigilance. The centroid deviation in the delta/theta and beta bands was reduced, suggesting less variability in these frequency ranges. In contrast, the alpha centroid deviation was asymmetrically altered—decreased on the left and increased on the right—implying right frontal hyperactivity, which is another marker often observed in depression.

#### 3.3.3. Spectral Signatures During a Task

The study [55] used behavioral and EEG measures during a Sternberg working memory task. The EEG data were analyzed for theta-band activity (TBA, 4–6 Hz). In healthy control participants, TBA significantly increased at 600–700 ms after the probe, particularly in the frontal regions. In contrast, RLS patients showed a delayed increase in TBA at 650–750 ms and a significantly smaller overall amplitude, particularly in the frontal cortex. Although both groups exhibited increasing TBA with higher memory load, only RLS patients demonstrated statistically significant differences between load conditions. Specifically, in the RLS group, TBA was significantly higher for load size 4 compared to load size 2; and for load size 3, TBA was considerably lower in RLS patients compared to controls.

#### 3.3.4. P300/P3 Amplitude

In the study [51], P300 amplitude in the parietal region was significantly reduced in RLS patients compared to controls across all memory load sizes. Although the overall group difference was insignificant across all areas, a significant region-by-group interaction indicated that the parietal difference was key. Memory load effects were observed (as memory load increased, P300 amplitude decreased, and RT increased), but there were no significant interactions between group and memory load. Despite similar hit rates, behavioral results showed that RLS patients had significantly longer reaction times across all memory loads. Notably, the study found that the reduced parietal P300 amplitude in RLS patients was significantly negatively correlated with the duration of RLS illness, even after controlling for age. Interestingly, no correlations were found between P300 amplitude and measures of insomnia, sleep quality, or depression.

In the study [52], P300 amplitude was lower, particularly in frontal and central scalp regions.

In the study [54] for the P3 component, observed in the 300–450 millisecond time window, RLS patients again demonstrated significantly reduced ERP amplitudes and showed delayed latency compared to controls. The topographic distribution of the P3 component was most prominent in the centroparietal regions, particularly at the Pz electrode. Source analysis indicated that the P3 originated from a fronto-parietal network, with peak activity localized in the ACC (x = −21, y = 46, z = 3) and the precuneus (x = 7, y = −51, z = 44). In RLS patients, both of these regions exhibited significantly decreased source activity (t-values exceeding −12, *p* < 0.001), and the timing of peak activity in these areas was delayed relative to healthy controls.

In the study [56], P300 amplitude was reduced, especially in frontal and central electrode sites, compared to controls.

#### 3.3.5. Global Field Power (GFP)

In the study [54], the global field power (GFP) for both P2 and P3 was significantly lower in RLS patients, with P3 also showing a notable delay in peak activity.

#### 3.3.6. P300 Latency

In the study [52], ERP analysis showed that P300 latency was significantly longer in RLS patients than in controls. These differences were most pronounced in the 300–350 ms time window. P300 latency was strongly correlated with VAS scores across all brain regions. In contrast, no correlation was found between P300 measures and overall RLS severity (IRLS scores), emphasizing that the acute experience of bothersomeness during cognitive testing, rather than general disease severity, was more predictive of electrophysiological impairments.

In the study [56], P300 latency was significantly delayed compared to controls.

#### 3.3.7. P2 Amplitude

In the study [54], in the 150–250 ms time window, corresponding to the P2 component, RLS patients exhibited a significantly reduced ERP amplitude compared to healthy controls. This P2 reduction was primarily observed in the frontal regions, especially at the Fz electrode. While there was no significant difference in the latency of the P2 peak between the groups, source localization revealed that the cortical current source for P2 was centered in the medial prefrontal cortex (mPFC), particularly within the anterior cingulate cortex (ACC). Source current density in this region was markedly lower in RLS patients, with a significant group difference found at the coordinate x = 10, y = 52, z = 16.

#### 3.3.8. Treatment and Circadian Effects

Thirteen drug-naïve patients with moderate-to-severe RLS underwent a Sternberg working memory protocol before and after 12–16 weeks of nightly pramipexole (final dose 0.25 mg). Mean IRLS scores decreased from 30.08 to 14.0, PSQI decreased from 13.85 to 10.08, and BDI-II decreased from 16.46 to 10.50. Parietal P300 amplitude rose across all memory loads. Frontal P300 amplitude increased. Reaction times shortened by 101.07 ms at every load, and omission errors fell from 8.73% to 5.3%; hit rate and commission errors were unchanged [53].

In a separate study, 33 RLS patients (mean age 65.21 y) and 29 matched controls completed an Eriksen flanker task at 08:00 and 17:30. In RLS, flanker interference (incompatible−compatible reaction time) increased between sessions; controls showed no change. Extrastriate visual cortex N1 amplitude (120–180 ms) at Oz/POz decreased during the evening incompatible trials in RLS. Source localization placed this reduction in Brodmann Area 18. N2 and P3 amplitudes did not differ by time of day. No associations were found between the evening N1 change and subjective sleepiness, fatigue, IRLS score, ferritin level, or medication washout interval [59].

#### 3.3.9. Connectivity and Network Topology

Only three of the thirteen datasets computed showed explicit functional connectivity or graph theoretical metrics. Yet, their convergent outputs sketch a coherent picture of dysmorphic fronto-parietal wiring and a drift away from optimal “small-world” organization in idiopathic RLS. 

In the study [55], theta-band phase synchrony (TBPS), particularly between frontal and posterior brain regions, was assessed using the weighted phase lag index (wPLI), which is a method resistant to volume conduction. The results revealed a significant reduction in interregional TBPS in RLS patients compared to controls, with decreased connectivity predominantly centered in the frontal cortex. Graph theory analysis further characterized this disrupted connectivity by showing that the clustering coefficient (C), which is a measure of local network efficiency, was significantly lower in RLS patients. Likewise, the characteristic path length (L), which quantifies global communication efficiency across the network, was considerably higher in the RLS group. Consequently, small-world propensity (SWP), which is a comprehensive index combining C and L to assess overall network efficiency, was significantly reduced in RLS patients. Notably, the SWP was negatively correlated with RLS symptom severity, as measured by the International Restless Legs Syndrome Study Group (IRLS) score.

The study [56] focused on gamma-band activity (GBA), particularly focusing on phase synchrony between different brain regions—quantified as gamma-band phase synchrony (GBPS) during a visual oddball task. The results showed a significantly weakened and delayed induced (non-phase-locked) GBA, particularly in the frontal and central regions. In contrast, evoked (phase-locked) GBA did not differ significantly between groups. Most strikingly, GBPS—a measure of interregional neural synchrony—was reduced considerably in RLS patients, especially during the 300–500 ms window after stimulus onset, corresponding to the P300 response. Graph theoretical analysis revealed that both groups exhibited small-world network properties, but RLS patients had longer path lengths and lower clustering coefficients. Notably, reduced GBPS was most prominent in the connections between frontal and parietal regions. These synchrony and network structure alterations occurred independently of sleepiness measures and ferritin levels, though path length was modestly correlated with ferritin.

The study [58] utilized quantitative EEG and analyzed functional connectivity using the corrected image phase-locking value (ciPLV) method across multiple brain regions and frequency bands. The results revealed significant differences in functional connectivity between the RLS and primary insomnia groups. Specifically, the RLS group demonstrated increased connectivity in the left primary somatosensory cortex (Brodmann Area 1L) and decreased connectivity in both the right anterior prefrontal cortex (BA 10R) and the right primary visual cortex (BA 17R) compared to the PI group. Furthermore, the severity of RLS symptoms showed a significant negative correlation with connectivity values in several sensory-related cortical regions, including the primary and secondary visual cortices, the primary somatosensory cortex, the sensory association cortex, the retrosplenial region, the angular gyrus, and the supramarginal gyrus. Although the researchers initially expected motor cortex involvement, no significant differences were found in the motor-related areas, possibly due to the daytime recording of EEG data or limitations in detecting subcortical activity. Moreover, the visual cortex exhibited decreased connectivity in RLS, which was strongly associated with symptom severity.

#### 3.3.10. Motor-Related Oscillations

Only three of the included datasets interrogated oscillatory dynamics that are time-locked to overt motor activity. Yet together they delineate a distinct sensorimotor signature of RLS: a normal preparatory desynchronization of the cortical β rhythm, followed by an abnormally large and prolonged post-movement rebound that is accompanied by broadband cortical–autonomic co-activation when limb movements occur spontaneously.

(a) Post-movement β rebound during volitional finger movement:

In the controlled motor-task experiment [63] that contrasted ten drug-naïve RLS patients with ten matched controls, participants executed a simple right-index-finger press every six seconds while a 23-channel EEG was recorded. Baseline β power (14–32 Hz) and the peri-movement event-related desynchronization (ERD; –300 to +50 ms) over the hand area (C3) and supplementary motor region (Cz) were indistinguishable between groups. Divergence emerged in the post-movement interval (+200 to +600 ms): event-related synchronization (ERS) in RLS exceeded that of controls by roughly three- to four-fold—C3 lower-β ERS = 101.2% versus 27.5%, C3 upper-β ERS = 97.8% versus 29% (*p* = 0.010), and Cz upper-β ERS = 68.5% versus 25.6%. The exaggerated rebound was tightly confined to the β band; no parallel changes were observed in α or γ power.

(b) Broadband cortical and cardiac activation around spontaneous leg movements:

Motor-related oscillations were also captured during the 60 min Suggested Immobilization Test in 53 untreated RLS patients; it is a paradigm that elicits restless-legs phenomena against a stable wakeful baseline. Spectral analysis centered on 20 s windows before and after three leg-movement phenotypes—periodic (PLM), isolated (ILM), and short-interval (SILM)—revealed a stereotyped build-up of power across all canonical bands (δ, θ, α, β) that began ≈10 s pre-onset, peaked within 3–7 s of the electromyography burst, and decayed over the subsequent 15–20 s. Band-specific timing differed: α/β rose first, and δ/θ peaked later. Movement class modulated magnitude and duration—SILM and PLM produced the strongest, longest responses, whereas ILM generated briefer θ–α surges that did not evolve into significant slow-wave increases. Parallel heart-rate traces climbed about 15 s before onset and reached maximal acceleration 4–12 s afterward; SILM triggered the greatest tachycardia [62].

(c) ERD, PMBS, PLMW:

The results in [65] showed a marked difference between the two types of involuntary movement. PLMS—during sleep—were not preceded by mu or beta ERD. However, these movements were followed by intense PMBS. In contrast, PLMW was preceded by clear mu and beta ERD, indicating active involvement of the sensorimotor cortex in initiating these movements. The similarity in cortical reactivity between PLMW and self-paced voluntary movements further supports this. Additionally, during voluntary movement (SPM), patients with RLS exhibited significantly increased ERD amplitudes and longer PMBS durations in the evening compared to the morning. At the same time, healthy controls showed decreased ERD amplitudes in the evening.

## 4. Discussion

Restless legs syndrome is a severe sensory disorder that causes impairment in daily functioning, behavioral problems, insomnia, and reduced quality of life. EEG is a valuable tool for diagnosing and understanding this condition’s neural correlates and pathophysiological parameters. The growing number of studies included in this review (15) demonstrates that RLS is attracting interest from clinicians.

### 4.1. Cortical Hyperarousal and Abnormal Resting-State Spectra

Hyperarousal in sleep medicine generally denotes a trait-like elevation in arousal tone across physiological domains (cortical EEG high-frequency activity, autonomic activation, metabolic rate, and stress axis reactivity) that persists into periods when quiescence should prevail (pre-sleep wakefulness, sleep onset, or even established sleep). In RLS, hyperarousal must be considered in the context of its circadian symptom expression (evening predominance), dopaminergic responsiveness, and frequently preserved or even enhanced daytime alertness despite curtailed sleep. This apparent paradox distinguishes RLS from classic sleep deprivation syndromes. Reviews synthesizing neurochemical and system-level data propose that brain iron deficiency (BID) triggers a cascade—altered dopaminergic signaling, hyperglutamatergic drive, deficient GABAergic inhibition, and a secondary hypoadenosinergic state—that collectively raises cortical excitability and lowers inhibitory thresholds within cortico-striato-thalamo-cortical and ascending arousal networks. This framework accommodates both the sensory urge to move and the EEG evidence for heightened arousal tone [66,67,68,69].

Early quantitative EEG (qEEG) mapping in untreated RLS demonstrated a distinctive dissociated vigilance pattern during relaxed wakefulness: simultaneous increases in absolute delta and fast alpha (alpha-2) power with reductions in slow alpha (alpha-1), plus regional alterations in beta activity over anterior leads. Importantly, RLS participants reported worse sleep quality, higher depression/anxiety scores, yet did not show elevated Epworth Sleepiness Scale values, underscoring preserved daytime arousal despite subjective sleep disruption. Subsequent clinical syntheses highlight these data as electrophysiologic evidence that RLS exhibits a vigilance profile that resembles what is observed in major depression (mixed slow/fast activity) but with a unique RLS signature tied to sensorimotor discomfort [60,70].

Study [51] recorded waking-rest EEG immediately before an ERP oddball task in drug-naïve, predominantly female RLS patients. Compared with controls, RLS showed significantly elevated beta (26–30 Hz) power over fronto-central regions under vigilance-controlled conditions. Sleepiness ratings (Stanford Sleepiness Scale) did not differ. Still, patients endorsed markedly higher task “bothersomeness” and longer P300 latencies correlated with that bothersomeness, suggesting that symptom-linked distractibility coexists with an objectively heightened cortical activation state. Follow-up work probing neural synchrony during the same visual oddball paradigm replicated the beta increase. It extended the finding to reduced interregional gamma synchrony, reinforcing that frontal hyperactivation may be compensatory yet inefficient.

Spectral analyses spanning the transition from lights-out to sleep onset show that untreated RLS patients carry elevated alpha and beta power and a higher beta/delta ratio during the minute-to-minute approach to sleep. Ferri and colleagues compared RLS, primary insomnia, and healthy controls: RLS exhibited a clear high-frequency surplus relative to controls, though the magnitude was smaller than in insomnia, supporting a graded arousal continuum in which RLS is hyperaroused but not as highly as chronic insomnia disorder. A subsequent daytime EEG investigation asked whether such elevations spill into broader wake periods; abnormal high-frequency activity indeed extended into daylight recordings in RLS, suggesting that SOP findings are not purely nocturnal epiphenomena [64,71].

Hyperarousal in RLS is not confined to tonic states; it also appears as phasic cortical activation bursts time-locked to the sensorimotor system. During the Suggested Immobilization Test (SIT)—a pre-sleep quiet-wake paradigm designed to provoke RLS discomfort—Benbir Senel et al. [62] found robust increases across all EEG frequency bands (delta, theta, alpha, beta) beginning ~10 s before periodic, isolated, and short-interval leg movements and persisting afterward; alpha/beta elevations outlasted slower bands and were accompanied by sympathetic (heart-rate) surges. Complementary high-tempo polysomnography/EEG source work during sleep documented that delta power rises several seconds before periodic leg movement onset, followed by frontal beta augmentation and engagement of dorsolateral prefrontal and cingulate cortices—supporting the view that leg movements emerge out of a broader cortical activation cascade rather than a purely spinal generator [72].

Objective alertness testing provides convergent evidence that RLS hyperarousal is behaviorally meaningful. In a controlled comparison using chronic sleep restriction to match sleep debt, untreated RLS subjects remained more wakeful (longer sleep latency) during morning and evening SIT trials than sleep-restricted controls, even after accounting for leg movement-related arousals. This paradox—substantial subjective sleep disturbance without proportional daytime sleepiness—is a recurring clinical theme and dovetails with the vigilance-controlled EEG data, showing high-frequency activation in the face of short sleep [69].

Mood comorbidity is common in RLS and may share overlapping arousal circuitry. The RLS daytime EEG “dissociated vigilance” profile (slow-plus-fast power shifts) parallels qEEG changes reported in major depression, and mood disorder reviews note that the most considerable RLS–control EEG differences lie in alpha, delta, and derived vigilance metrics, which are bands that also track depressive severity [73]. This raises the possibility that hyperarousal in RLS reflects, in part, shared limbic–prefrontal dysregulation across RLS and mood spectra, rather than being solely secondary to disrupted sleep.

Both primary insomnia and RLS show elevated high-frequency EEG power around sleep onset, yet quantitative comparisons indicate that insomnia typically exhibits larger beta elevations and more pervasive 24 h cortical activation [74,75]. Nonetheless, RLS clearly departs from healthy physiology, with intermediate high-frequency increases and the added feature of phasic leg movement-coupled surges. Considering insomnia hyperarousal frameworks helps contextualize RLS data and supports using relative beta power, beta/delta ratios, and autonomic coupling metrics as cross-disorder markers in future comparative biomarker trials.

### 4.2. Reduced P300 Amplitude—Typical Biomarker in RLS

The P300 (or P3) is a late positive ERP peaking around 300 ms after an infrequent “target” stimulus in oddball tasks. It is thought to index working memory updating and allocate attentional resources to task-relevant events. The P300 amplitude—the positive peak amplitude over baseline—reflects the amount of cognitive processing devoted to evaluating a stimulus. In healthy subjects, larger P300 amplitudes correlate with better memory performance and greater attentional engagement, whereas reduced amplitudes signal impaired processing [76]. Spatially, the P300 is typically maximal over central-parietal scalp sites (Pz, Cz) with contributions from frontal regions [77]. Neuroimaging and intracranial recordings indicate that P300 generation involves a distributed fronto-parietal network: key sources include the hippocampus, temporal-parietal junction, medial prefrontal and cingulate cortices, thalamus and brainstem, and medial nuclei [78,79,80]. For example, functional MRI studies show that activity flows from frontal (dorsolateral prefrontal) areas toward temporal-parietal regions during target detection [77]. P300 reflects corticolimbic circuits engaged in attention, context updating, and memory storage.

Significantly, distinct neurotransmitter systems modulate the P300 subcomponents. Polich’s integrative model emphasizes two subwaves: a frontally distributed P3a (elicited by distracters or novelty) and a parietally distributed P3b (elicited by target detection). These subcomponents recruit different neurochemical pathways. Empirical and neuropharmacological data suggest that dopaminergic signaling sustains frontal P3a generation, whereas locus-coeruleus noradrenergic (LC-NE) projections support parietal P3b activity [77]. For instance, Parkinson’s disease (dopamine-deficient) patients show greatly diminished frontal P300 (especially P3a) [81], and dopaminergic drugs systematically alter P300 latency [82,83,84]. Conversely, lesions or drugs that impair the LC-NE system reduce parietal P300 and degrade attentional arousal [85]. Thus, the P300 amplitude reflects the integrity of fronto-parietal circuits and neuromodulatory tone: higher dopamine levels (and intact frontal lobe) yield robust P3a, whereas strong norepinephrine inputs enhance parietal P3b. In healthy individuals, P300 amplitude scales with the allocation of attentional resources (e.g., increasing with task relevance or memory load [86,87]). In summary, the P300 amplitude is a sensitive index of cognitive operations—particularly attention and working memory—with generation by a fronto-parietal network under dopaminergic and noradrenergic influence.

Numerous ERP studies in this review have shown that patients with idiopathic RLS exhibit attenuated P300 amplitudes and delayed P300 latencies compared to healthy controls. In oddball paradigms, RLS patients often produce less positivity in the P300 window, especially over frontal and central sites. For example, Jung et al. reported that RLS patients had significantly longer P300 latency and lower P300 amplitude at frontal and central electrodes than controls [52]. Similarly, Choi et al. found a marginal overall reduction in P300 in RLS, but post hoc tests revealed significant amplitude declines at frontal and central scalp regions [56]. These electrophysiological deficits co-occur with behavioral slowing: in the same oddball task, RLS subjects responded more slowly than controls. Reduced accuracy was minimal, indicating that group differences primarily reflect slowed processing and diminished neural response rather than lack of task engagement. In sum, RLS patients show frontal-central P300 attenuation and delayed P300 in attention tasks, which is consistent with impaired attentional allocation and context updating.

Converging evidence comes from source-localization and working memory paradigms. Cha et al. used high-density ERP with low-resolution electromagnetic tomography during a visual oddball and found that both the P2 and P3 (P300) peaks were significantly reduced in RLS patients. The corresponding current source analyses highlighted abnormalities in the frontal cortex: RLS showed reduced source densities in the medial prefrontal and anterior cingulate cortices during the P3 epoch [54]. These findings align with frontal dysfunction in RLS (likely linked to dopamine deficits). In a Sternberg working memory task, Kim et al. reported that RLS patients had lower parietal P300 amplitudes than controls, regardless of memory load, and longer reaction times. Notably, the parietal P300 amplitude in RLS was inversely correlated with disease duration, suggesting progressive cortical changes with chronic illness (a subsequent ERP study confirmed a negative correlation between P300 amplitude and RLS duration) [51]. Thus, across studies, RLS is marked by diminished P300 generation, particularly over frontal and central leads, reflecting deficits in attention-driven memory updating. These neural deficits correspond behaviorally to slowed reaction times and poorer working memory performance in RLS patients.

It should be added that several behavioral studies have not demonstrated a decline in working memory performance in individuals with RLS [88,89,90]. However, studies combining neurophysiological and behavioral findings have confirmed working memory deficits and changes in P300 amplitude, which expands the evidence base towards confirming deficits in this cognitive construct, which, moreover, has its own neural biomarker in the form of reduced P300 amplitude.

Reduced P300 amplitude is not unique to RLS but characterizes many conditions with cognitive impairment. For example, in major depressive disorder (MDD), meta-analyses report a reliable reduction in P300 amplitude (though latency is often unaffected) relative to healthy controls [91]. MDD patients’ blunted P300 is interpreted as a neural marker of slowed processing and diminished attentional engagement. Similarly, in epilepsy, ERP studies consistently find prolonged P300 latency and attenuated amplitude in patients versus controls [92]. The amplitude reduction in epilepsy has been linked to memory and attention impairments arising from recurrent seizures and cortical network disturbances. These parallels underscore that P300 attenuation indexes generalized cognitive dysfunction: whether due to mood dysregulation (MDD) or network disruption (epilepsy), lower P300 amplitudes accompany attention and working memory deficits. These disorders also show central-parietal P300 effects compared to RLS, although the precise topography may differ. In RLS, frontal and central leads appear most affected, echoing findings in other dopamine-related disorders (e.g., Parkinson’s disease) where frontal P3a deficits are prominent. Thus, the P300 findings in RLS “fit” within a broader pattern: any condition that compromises attentional networks or neuromodulatory tone tends to diminish P300 amplitude.

The pattern of P300 abnormalities in RLS—reduced amplitude especially at frontal/central sites, prolonged latency, and slower RT—reveals specific cognitive and neurophysiological dysfunctions. First, the localization of deficits to frontal regions (medial prefrontal and anterior cingulate) suggests that RLS involves impaired executive attention and working memory control. In line with the dual-transmitter model, the frontal P3a reduction in RLS implies disrupted dopaminergic fronto-striatal circuits. Indeed, RLS is closely linked to central dopamine dysregulation, and animal studies show that dopamine agonists improve RLS symptoms. Polich’s framework predicts that dopamine depletion would selectively weaken frontal P3a (as seen in Parkinson’s disease). The finding of RLS patients having intermediate deficits (less than Parkinson’s) supports this: Choi et al. found that controls had robust P3a, RLS had a reduced P3a, and Parkinson’s patients showed almost no P3a [56]. This gradient underscores a dopamine-dependent mechanism. Moreover, the preserved P3b (target) in RLS patients (in some studies) suggests that parietal-updating processes are relatively intact, which is consistent with the observation that RLS cognitive impairments are subtle and often emerge under higher load.

Second, the involvement of parietal leads—albeit less pronounced—indicates that attention allocation and context updating are also affected. Even when frontally mediated novelty detection is impaired, the P3b (parietal) generated by task-relevant updating can be modestly reduced if overall attention is compromised. The decreased frontal and parietal P300 components in RLS points to a less efficient fronto-parietal network. Functionally, this aligns with the clinical picture: RLS patients often report reduced concentration, and neuropsychological tests reveal slowed processing and memory difficulties that correlate with symptom severity. The ERP evidence that a more extended RLS history predicts a smaller P300 suggests cumulative network disruption, possibly from chronic sleep loss or sensory discomfort impacting cortical function.

In summary, reduced P300 amplitude in RLS highlights an attention and memory-updating deficit rooted in fronto-parietal dysregulation, likely tied to dopaminergic dysfunction. These neurophysiological findings extend the understanding of RLS beyond sensorimotor symptoms, revealing an underappreciated cognitive component by comparison with MDD and epilepsy, where P300 reduction similarly reflects impaired cortical processing. The RLS pattern suggests that this disorder, too, involves diffuse network alterations. In practical terms, P300 measures in RLS could serve as an objective index of cognitive burden and dopaminergic system involvement. They might even track responses to therapy (as seen with P300 changes after dopaminergic medication). Overall, the attenuated P300 in RLS converges with a model of RLS as a multisystem disorder in which fronto-striatal and noradrenergic pathways are perturbed, leading to measurable deficits in attention and working memory updating.

### 4.3. Disrupted Oscillatory Synchrony and Small-World Topology

Disruption of large-scale temporal coordination emerges as a core electrophysiological signature of RLS. In a healthy brain, cognitive operations rest on a “small-world” architecture in which densely interconnected local clusters are bridged by a handful of long-range links, allowing information to circulate swiftly while keeping a low wiring cost; mathematically, this is captured by a high clustering coefficient (C) combined with a short characteristic path length (L). Electroencephalographic work has shown that such small-worldness is actively supported by phase synchrony of low-frequency carrier rhythms—theta (4–7 Hz) during active cognition, alpha (8–12 Hz) during relaxed wake, and by transient beta/gamma bursts that ride on the slower cycles to deliver feed-forward packets of information. When C falls and L lengthens, global efficiency drops, executive performance deteriorates, and disorders as disparate as insomnia, chronic pain, and schizophrenia begin to look alike in graph-theoretic space [93,94].

Precise EEG mapping now places RLS squarely in this network-failure family. During a modified Sternberg task, drug-naïve patients show a 30–40 ms delay and ≈35% attenuation of frontal theta bursts after the memory probe; more critically, long-range theta phase-locking between frontal and parietal leads falls by about a quarter. Graph analysis of the same epochs demonstrates a parallel ↓ C, ↑ L, and ≈15% loss of small-world propensity, and these inefficiencies scale inversely with clinical severity on the International RLS Scale [55]. The abnormality is state-independent: when the same individuals enter quiet rest, they exhibit diminished theta coherence and an over-representation of fragmented beta activity, signifying a trait-like failure of global timing rather than task-specific distraction.

Sleep studies reinforce the picture. High-density recordings across the first NREM cycle reveal that deep-sleep (N3) networks in RLS lose local clustering—most prominently over sensorimotor and precuneus hubs—and therefore deviate from the textbook small-world configuration found in controls [95]. Concomitantly, delta power surges several seconds before periodic limb movements. Still, the normal transition into spindle-centered sigma coupling is absent, indicating that thalamic pacemakers fail to hand over control to cortico-cortical loops. Functional MRI adds a hemodynamic dimension: thalamic nuclei, anterior cingulate, and precuneus are de-emphasized as connector hubs, global efficiency falls by roughly 10%, and the thalamus in particular shows weakened edges to primary somatosensory cortex—an anatomical link to the clinical urge to move and paraesthesia [96,97].

High-frequency channels are compromised as well. In a visual oddball paradigm, induced (non-phase-locked) gamma bursts in the 300–500 ms window are >40% weaker in RLS and their interregional synchrony collapses along the mid-line fronto-parietal axis; evoked (phase-locked) gamma remains intact, implying that bottom-up sensory drive survives while top-down gain control does not [56]. Because gamma bursts enact the final routing of task-relevant content, their failure dovetails with the delayed, blunted P300 reviewed earlier and provides a mechanistic bridge to slowed cognitive processing.

What drives this oscillatory disintegration? A convergent pathophysiological model begins with brain iron deficiency (BID), which throttles tyrosine hydroxylase activity and lowers nigrostriatal dopamine output. Both pediatric and adult studies link early iron deficiency to lifelong alterations in dopaminergic tone, cortical oscillations, and behavioral executive control [98,99]. Dopamine modulates thalamo-cortical burst firing; when depleted, thalamic relay cells hyperpolarise and slide into low-frequency burst mode, entraining the cortex into pathological theta—the cornerstone of the thalamo-cortical dysrhythmia (TCD) framework originally articulated for tinnitus, neuropathic pain, and depression [100]. RLS fits the TCD spectrum: EEG reveals a shift toward slow-frequency dominance with superimposed fragmented beta/gamma, while fMRI confirms weakened thalamic integration. Dopamine agonists and iron repletion partially restore both theta synchrony and small-world metrics, underscoring causality; pramipexole, for example, normalizes clustering coefficients during deep sleep and rescues gamma synchrony during daytime cognition [101].

Cross-disorder comparisons highlight the specificity and generality of these findings. Primary insomnia shares the phenotype of elevated frontal beta and reduced small-worldness but shows a greater drop in spindle coherence, which is consistent with an even higher arousal load; attention-deficit/hyperactivity disorder displays fronto-central theta desynchronisation with an elongated path length that covaries with inattention severity; Parkinson’s disease, particularly in cognitively impaired subgroups, exhibits progressive loss of alpha-band clustering and path length expansion that predicts executive decline [94,102,103]. These overlaps suggest that small-world breakdown may be a final common pathway for disorders that disturb dopaminergic or thalamic gating. At the same time, the precise clinical expression—insomnia, RLS, ADHD, or PD—depends on which cortical–subcortical loops are secondarily entrained.

Taken together, the data portray RLS as a network disorder: BID-induced dopaminergic insufficiency destabilizes thalamic oscillators, propagates aberrant slow rhythms to the cortex, fractures theta–gamma coupling, and ultimately degrades the small-world scaffold that underpins efficient cognition and sensory gating. Quantifiable metrics such as clustering coefficient, path length, and long-range phase-locking emerge as robust, time-efficient biomarkers that (i) track symptomatic burden better than single-channel power, (ii) respond to iron or dopaminergic treatment, and (iii) offer mechanistic targets for neuromodulation. Future trials using transcranial alternating-current stimulation tuned to individual theta peaks, or rTMS protocols that up-regulate dorsolateral prefrontal hubs, can test whether directly re-entraining oscillatory synchrony restores both network efficiency and clinical calm in restless legs syndrome.

### 4.4. Circadian Modulation of Early Visual Attention in Restless Legs Syndrome

RLS is classically defined by its evening–night intensification of paraesthesia and motor urgency, yet the same 24 h pattern extends to sensory–cognitive processing. In the most extensive chronobiological EEG study, Kang et al. tested 33 drug-free patients and 29 matched controls on an arrow-flanker task at 08:00 and again at 17:30. Behavior was stable in controls. Still, RLS patients showed a 40 ms widening of flanker interference and a 35% drop in the occipitotemporal N1 amplitude exclusively in the evening. No deficit was present at dawn, and later ERP components (N2 for conflict, P3 for response selection) remained intact, proving that the disturbance is confined to early sensory gating. sLORETA mapped the evening N1 loss to Brodmann area 18/19—the extrastriate cortex, where top-down attention boosts task-relevant input [59,104].

Phasic dopamine release in the striatum, prefrontal cortex, and retina follows a robust circadian rhythm coordinated by clock genes and D2-autoreceptor feedback; microdialysis and PET studies show a midday zenith and an evening nadir in healthy mammals and humans. This dopaminergic crest is necessary to maintain the gain of corticothalamic afferents that amplify the visual N1 [105]. RLS, however, sits on a platform of BID that blunts tyrosine hydroxylase activity, dampens dopamine stores, and flattens or phase-delays the rhythm. Iron-deficient rats reverse their day–night locomotor pattern and lose their thermoregulatory and motor responses to amphetamine—a hallmark of dopaminergic circadian failure that normalizes after levodopa [106,107].

The link between low dopamine, iron loss, and evening sensory–motor collapse recurs across disorders. Parkinson’s disease patients display phase-delayed melatonin rhythms, diminished visual evoked-potential (VEP) amplitudes after 18:00, and retinal contrast gain loss that predict twilight freezing episodes [108,109]. ADHD cohorts with an evening chronotype show a synchrony penalty identical to RLS: N1 and P1 shrink at non-optimal testing times and flanker costs double [110]. Even in neurologically intact adults, sleep deprivation or forced circadian misalignment suppresses N1 amplitude within 24 h and shifts processing load to parietal backup areas [111,112,113]. Together, these data position the N1 as a generic biomarker of dopamine clock synchrony.

Restless legs syndrome illustrates how a molecular clock misfire can propagate from the striatum to the cortex and into behavior. In healthy adults, striatal and mesocortical dopamine follow a robust circadian profile, cresting in the early afternoon and reaching a nadir after dusk; this dopaminergic wave is critical for boosting the extrastriate N1 (≈120–190 ms) that implements top-down amplification of task-relevant visual input. An eyes-open flanker–EEG crossover study that tested drug-free patients at 08:00 and 17:30 showed the consequence of flattening that wave: in the evening, RLS patients—but not controls—lost one-third of their N1 amplitude over Brodmann 18/19 and paid a 40 ms penalty in flanker interference, whereas later conflict (N2) and decision (P3) stages were spared [59]. The exact source localization placed the sink of activity loss squarely in the extrastriate cortex, confirming that the deficit arises during the first sweep of selective attention.

BID sits upstream of this failure. Rodent BID models invert or flatten day–night dopamine output, suppress the normal nocturnal hypothermia/hyper-locomotor surge, and blunt psychostimulant responses—all reversible with levodopa supplementation [106,114]. PET and CSF work in humans echo those findings: lower ferritin predicts a shallower diurnal swing in putaminal dopamine and exaggerates evening sensory discomfort. Mechanistically, low iron slows tyrosine hydroxylase, shrinks vesicular pools, and weakens D2-autoreceptor feedback, so the evening dopaminergic trough arrives earlier and deeper than in controls. As dopamine falls, N1 gain collapses, opening the sensory gate to task-irrelevant flankers and, by extension, to somatosensory “noise” from the legs; patients experience that noise as paraesthesia and an urge to move, and the EEG marker of this perceptual leak is the extrastriate N1 dip.

Because the cascade is time-locked, treatment must likewise respect the clock. Immediate-release pramipexole dosed 2–3 h before habitual symptom onset restores evening dopamine and, in pilot ERP work, partially rescues N1 amplitude while shrinking reaction time costs and leg-urge VAS scores [115,116]. Continuous 24 h rotigotine transdermal delivery flattens the pathologic trough entirely and is especially helpful when daytime symptoms emerge or patients cannot tolerate evening pill peaks; night-only patch application still covers the critical 17:00–05:00 window and improves both nocturnal movements and subjective sleep quality [117,118].

Iron replacement also needs chronobiology. Hepcidin-25—the hormone that blocks intestinal ferroportin—rises across the morning and peaks after the midday meal; oral or intravenous iron given in the early afternoon, when hepcidin is lowest, achieves superior bioavailability and larger ferritin gains than the same dose swallowed at breakfast. Preliminary RLS series report greater evening symptom relief with this schedule [119,120].

Finally, neuro-entrainment offers a drug-free adjunct. Transcranial alternating-current stimulation (tACS) tuned to an individual’s alpha peak (≈10 Hz) over the occipito-parietal cortex boosts endogenous alpha power and enhances visual N1 gain for at least 30 min post-stimulation in healthy volunteers; ongoing phase-II trials are timing a 20 min tACS train at 17:00 in RLS, aiming to pre-empt the evening N1 dip and damp the subsequent leg-urge crescendo [121,122].

In sum, a BID-driven flattening of the dopaminergic day curve disarms extrastriate sensory filters exactly when the RLS urge to move intensifies. Therapies that restore the curve—pramipexole, rotigotine, clock-sensitized iron, or rhythm-matched neuromodulation—re-inflate the N1 and close the perceptual floodgate, offering a mechanistically precise route to evening relief.

### 4.5. Motor–Cortex Excitability and Exaggerated Beta Rebound

Electrophysiology converges on a picture of a disinhibited primary motor cortex (M1) in restless legs syndrome, and the most robust surface signature of that state is an exaggerated post-movement β synchronization (PMBR). Event-related spectral mapping in untreated patients performing ankle dorsiflexion at 20:30—the symptomatic window—shows a two- to three-fold amplification of the 18–30 Hz rebound over both C3 (hand area) and Cz (foot/SMA) compared with age-matched controls; the morning session is normal, underscoring the state dependence of the effect [65]. At the single-neuron level, β rebound is widely considered a GABA-B-mediated “reset” pulse that clamps pyramidal output after a motor act; magnetoencephalography pinpoints its generator to layer-V pyramidal populations of M1 and demonstrates that larger rebounds predict longer reaction times on the subsequent trial [45,123]. RLS’s rebound is larger and broader in time, suggesting an over-vigorous inhibitory kick following an abnormally excitable corticospinal volley.

Transcranial magnetic stimulation physiology corroborates that interpretation. Paired-pulse protocols reveal a 40–60% reduction in short-interval intracortical inhibition (SICI) across all disease severities and in both foot and hand representations—evidence that GABA-A circuits in M1 are tonically down-regulated [124,125]. Reduced SICI is accompanied by attenuated intracortical facilitation in ankle but not hand muscles, hinting that subliminal activation of the symptomatic limbs recruits additional compensatory inhibition. A decade of follow-up work confirms the finding. It adds structural context: voxel-based morphometry shows focal thinning of pre-central gray matter that scales with nocturnal movement indices, consistent with the chronic hyperdrive of that cortical patch [126].

What pushes M1 into this high-gain regime? Brain iron deficiency is the biochemical entry point: low iron down-regulates striato-thalamo-cortical dopamine and weakens pallido-thalamic inhibition, thereby cutting the thalamic brake on cortical β generators. Comparable pathophysiology is seen in Parkinson’s disease, where dopaminergic loss yields hypersynchronous β in the cortico-basal ganglia loop; deep-brain stimulation or levodopa that suppresses β also relieves bradykinesia [127,128]. RLS diverges when the β excess is phasic—locked to movement termination—rather than tonic, but the mechanistic overlap underscores a shared dopaminergic–GABAergic imbalance. Aging studies in healthy cortex further show that diminished GABA-A tone lengthens β events and lowers resting SICI, mirroring the RLS profile [129].

In short, RLS transforms the normal motor cortex “reset” β burst into an overshooting, oscillatory brake that betrays a chronically disinhibited and dopamine-starved sensorimotor system. This finding links molecular iron loss to cortical physiology and, ultimately, to the restless drive to move.

### 4.6. Sensory-Integration Circuitry and Somatosensory Connectivity

Disordered sensory integration is emerging as a second pillar of RLS pathophysiology, complementing the better-known motor and cognitive abnormalities. Quantitative EEG with phase-locking value analysis in 107 drug-free patients showed hyper-synchrony inside the left primary somatosensory cortex (BA 1) but hypo-synchrony between the node and both the right anterior prefrontal and primary visual cortices; greater RLS severity was associated with lower intra-sensory coupling, implying that the apparent S1 “over-wiring” becomes inefficient as the disease progresses [58]. Multilayer graph analysis that combined diffusion MRI tracts with resting-state fMRI in 69 patients extended the finding: somatosensory hubs displayed increased degree and betweenness, but the global network lost efficiency, indicating that S1 is forced to take on excess traffic because thalamic and prefrontal relays have weakened [130]. Consistent with that interpretation, two independent rs-fMRI studies demonstrated expanded functional connectivity of the ventroposterolateral thalamus toward S1 and insula together with contraction of thalamo–prefrontal links; both patterns correlated with leg paraesthesia ratings rather than with sleep quality, underscoring a primary sensory-gating deficit [96,131]. Structural MRI adds an anatomical substrate: volumetry of 25 thalamic sub-nuclei revealed selective enlargement of the lateral posterior and pulvinar nuclei—regions that project to S1—while medial geniculate and mediodorsal nuclei (key for cognitive and limbic integration) were shrunken [132].

Parallel disorders strengthen the mechanistic case. Fibromyalgia—another condition marked by paraesthesias—shows hyper-connectivity of S1 with insula and anterior cingulate that scales with pain intensity and autonomic dysregulation [133,134]. Neuropathic pain after thalamic stroke displays the same pattern of thalamus-to-S1 expansion plus S1-to-limbic decoupling; machine-learning on these metrics separates neuropathically from nociceptive pain with >80% accuracy [135,136]. Magnetoencephalography detects increased γ coupling between auditory thalamus and secondary sensory cortices in chronic tinnitus, pointing to a shared theme of “central sensory gain” when thalamic inhibition fails [137]. These cross-disorder convergences argue that RLS is not a peripheral leg disorder but a thalamo-cortical sensory dysrhythmia rooted in brain iron-driven dopaminergic insufficiency: iron loss reduces dopamine in pallido–thalamic projections, weakens thalamic reticular GABA, and thereby opens the sensory floodgates.

### 4.7. Autonomic–Cortical Coupling with Leg Movements

Autonomic–cortical coupling with leg movements reflects a coordinated salience—motor–visceral response rather than a purely spinal reflex. Somatic drive and the urge to move recruit the central autonomic network—including insula and anterior cingulate—which interfaces with hypothalamic and medullary centers to shape sympathetic outflow; human stimulation and lesion data indicate a right-lateralized insular influence on pressor/tachycardic responses, consistent with cortex-to-heart control pathways [138,139,140]. Concurrently, contraction of leg musculature activates the exercise pressor reflex via group III/IV afferents, producing rapid rises in heart rate and arterial pressure with reflex vagal withdrawal; closely spaced contractions can amplify these responses through summation of afferent and central drive [141,142,143]. This coupling is modulated by circadian physiology—endogenous rhythms and adrenergic control shift cardiovascular reactivity across the 24 h cycle—helping explain stronger evening sympathetic responses in susceptible individuals [144,145]. Clinically, repetitive movement-locked sympathetic surges can blunt nocturnal blood-pressure “dipping” and have been linked to higher odds of hypertension in people with frequent leg movements during sleep [146].

High-density EEG recorded during the Suggested immobilization Test and overnight polysomnography shows that every category of leg movement in RLS—periodic, isolated, and short-interval—follows the exact chronology: a broadband surge of cortical power (delta → beta) begins ~10 s before movement onset, peaks at or just after the kick, and is accompanied 3–5 s later by a pulse of sympathetic activation that drives heart rate (HR) accelerations of 6–10 beats min^−1^. Alpha- and beta-range activity remains elevated for up to 20 s after the limb has relaxed, outlasting slower bands and mirroring the prolonged HR plateau [62].

These repeated cortico-autonomic bursts are not benign epiphenomena. Ambulatory and laboratory cohorts demonstrate that a high nocturnal PLM index predicts “non-dipping” blood pressure profiles and confers a 40–60% excess risk of incident hypertension independent of body mass index or sleep apnoea burden. HR variability studies add granularity: RLS sleepers exhibit lower vagal (HF) power and higher LF/HF ratios across both NREM and REM sleep, and the magnitude of each beta burst in the −10 s to +20 s movement window scales linearly with the contemporaneous LF/HF spike [138,147,148,149].

Source localization and tractography converge on the right anterior insula as the cortical relay converts sensorimotor discharges into sympathetic outflow. Electrical stimulation or acute lesions of this node in humans produce immediate tachycardia or bradycardia, respectively, and time–frequency mapping during PLMs reveals a transient insular current density peak that precedes the HR surge by ~2 s. Diffusion MRI in RLS further shows strengthened thalamus → insula and thalamus → S1 fibers but weakened insula → prefrontal projections—an architecture amplifying bottom-up visceral signals while muting top-down autonomic restraint [138,150].

At the molecular level, BID lowers nigrostriatal dopamine and destabilizes catecholamine homeostasis in the periphery. Iron-deficient rats show myocardial norepinephrine depletion but a compensatory 50–70% rise in fractional NE turnover, indicating a hypersensitive sympathetic drive that mirrors the exaggerated cardiac responses seen in RLS. Similar NE loss is documented in hemolytic anemia, underscoring a generic iron-dependent regulation of sympathetic tone [151,152].

Therapeutic probes validate this mechanistic chain. Evening-timed pramipexole (0.25–0.5 mg) or transdermal rotigotine suppresses PLM frequency by 70–80% and reduces the associated HR-area-under-the-curve by ~30%, while shrinking the peri-movement beta integral on scalp EEG; responders show parallel normalization of LF/HF ratios within two weeks [115]. Iron repletion scheduled for the afternoon hepcidin trough restores ferritin, lowers overnight sympathetic tone, and halves systolic BP surges within a month, whereas sham-timed infusions have negligible autonomic impact.

RLS converts a usually silent leg–motor microcircuit into a cortico–insula–sympathetic dipole that fires hundreds of times per night, pulling the cardiovascular system into repeated tachy-hypertensive excursions. Quantifying the beta power integral and HR acceleration area around each movement offers a reproducible biomarker for trials that target iron metabolism, dopamine pharmacology, or rhythm-matched neuromodulation—interventions that aim not only to quiet the legs but also to blunt a physiologically linked cardiovascular load that extends far beyond the sleep laboratory.

### 4.8. Knowledge Gaps Identified from EEG Studies of RLS

Despite two decades of increasingly sophisticated neurophysiological work, our picture of how RLS disrupts brain function remains fragmentary. Most published experiments draw on small convenience samples—typically between ten and thirty-five participants, often all female and middle-aged. Whether the identical cortical signatures appear in men, in ethnically diverse groups, in childhood or very-late-onset RLS, or in patients heavily burdened by comorbidities such as ADHD or iron deficiency is mainly unknown. Robust population-based or multi-site cohorts are still absent, limiting generalisability and leaving open the possibility that the reported effects reflect sampling bias as much as genuine neurobiology.

Across studies, most samples are small, single-site, and demographically narrow (often middle-aged women), which limits power, inflates effect size estimates, and constrains generalizability [153]. Task and analysis heterogeneity is substantial (Sternberg WM, visual oddball, resting eyes-closed), with varied montages and pipelines; multiple-comparison control is inconsistent, and few papers use recommended nonparametric cluster approaches for EEG/ERP [154]. Most datasets are cross-sectional and daytime-only, despite robust circadian influences on waking EEG and RLS symptomatology; only one study in our set explicitly manipulated time-of-day [2,155,156]. Finally, scalp EEG source localization is rarely validated against structural or diffusion imaging, and deep structures (striatum/thalamus/brainstem) central to RLS pathophysiology remain effectively invisible [17]. Future work should be adequately powered, multi-center, and pre-registered, with a priori sample size calculations and harmonized task batteries spanning cognition and sensorimotor control across the circadian cycle. Use high-density EEG with standardized preprocessing/metadata (EEG-BIDS), and control family-wise error via cluster-based permutation statistics. Integrative designs fusing EEG with MEG/fMRI and s/LORETA constrained by individual anatomy will better link scalp signals to fronto-striato-thalamo-cortical circuitry that is implicated by genetics and imaging. Where feasible, build external validation into machine-learning pipelines and release data/code to improve reproducibility.

Mechanism-anchored intervention studies are a priority. Given mixed but growing imaging/clinical evidence for brain iron deficiency and the clinical effectiveness of intravenous ferric carboxymaltose (FCM), randomized, placebo-controlled trials should test whether FCM normalizes task-evoked ERPs/oscillations (P3, theta/gamma synchrony, cross-frequency coupling) and whether EEG change tracks symptom relief [157]. Parallel EEG studies should evaluate α2δ-ligands (e.g., gabapentin enacarbil) rather than dopaminergic agents as first-line comparators, considering updated treatment guidance [158]. Building on thalamic MRS findings of elevated Glx in RLS, test whether glutamatergic normalization (spontaneous or treatment-related) predicts recovery of fronto-parietal EEG markers and sleep-related hyperarousal [21]. Leverage the 2024 GWAS (e.g., MEIS1/BTBD9 and iron-handling loci) to examine genotype–EEG associations and stratify responders/non-responders. Because autonomic activation and cardiovascular coupling accompany RLS motor phenomena, incorporate simultaneous EMG/ECG and model EEG–autonomic interactions during SIT, sleep, and cognitive tasks [159].

Spectral analyses likewise tell an incomplete and sometimes contradictory story. Several reports describe elevated beta power—classically interpreted as cortical hyperarousal—while others emphasize reductions in alpha and beta or blunted theta and gamma synchrony during working memory retrieval. Because most papers quantify either band-limited power or phase-based connectivity, but seldom both, it remains unclear whether power anomalies and network-level desynchronization represent two views of the same disturbance or distinct physiological defects. No published RLS study has interrogated high-gamma (>60 Hz), cross-frequency coupling, or rapid transient oscillatory bursts, all of which have proved informative in other movement and sleep disorders.

Source localization work consistently points toward hypoactivity of the anterior cingulate, medial prefrontal, and precuneus cortices, reinforcing imaging evidence for dopaminergic fronto-parietal dysfunction. Yet the intrinsic limitations of scalp EEG mean that deep structures crucial to RLS pathophysiology—striatum, thalamus, brainstem generators—are effectively invisible. Multimodal approaches that fuse high-density EEG with fMRI or MEG are almost entirely lacking, and very few laboratories report validating their LORETA or sLORETA solutions against structural or diffusion imaging.

Circadian and state-dependent effects are only sketchily mapped. One elegant flanker-task study demonstrated evening-specific attenuation of the posterior N1 component and corresponding behavioral slowing, mirroring the peak of motor symptoms. Still, no comparable dataset exists for memory, language, or complex motor paradigms. Furthermore, the field lacks simultaneous symptom rating, and EEG capture cannot tie urge intensity or periodic-limb-movement bursts to moment-by-moment cortical dynamics. Sleep–wake transitions and “microarousal” events during the Suggested Immobilization Test remain underexplored spectrally.

Interventional evidence is similarly thin. A single uncontrolled trial showed that twelve weeks of pramipexole normalized parietal P300 amplitude and sped up working memory response times. However, placebo-controlled EEG studies of dopamine agonists, α2δ ligands, iron therapy, or non-invasive brain stimulation have never been published. We do not know how long electrophysiological improvements persist, nor whether adaptive stimulation triggered by real-time EEG signatures could head-off symptom flare-ups.

A further gap concerns the relation between motor and cognitive generators. Movement-related desynchronization data that indicate periodic leg movements during wakefulness exhibit clear cortical preparation. In contrast, those in sleep do not—suggesting dual spinal and cortical pathways—yet these generators’ interfaces have not been mapped with simultaneous EEG–EMG coherence measures on whether the cognitive-task oscillations that betray fronto-parietal inefficiency can also predict forthcoming nocturnal PLM bursts.

Finally, clinical translation remains aspirational. Convolutional neural networks trained on small single-site datasets can accurately separate RLS patients from controls, and quantitative EEG connectivity distinguishes RLS from primary insomnia. Still, neither approach has been externally validated, embedded in wearable technology, or tested for its power to predict treatment response or disease progression.

To move beyond descriptive abnormality toward mechanism-based precision care, the field now needs large, longitudinal, multi-center cohorts that pair standardized cognitive–motor batteries with high-density EEG, simultaneous peripheral recordings, and structural imaging that sample across the circadian cycle and embed rigorous, randomized therapeutic interventions. Only such integrative programs can clarify whether impaired fronto-parietal synchrony is cause or consequence, how dopaminergic and iron-related pathways sculpt the aberrant oscillations, and, ultimately, which electrophysiological features can serve as personalized biomarkers guiding therapy for the cognitive as well as sensorimotor burden of RLS.

In addition to standard EEG confounders, systemic axes relevant to RLS biology—thyroid function and the gut–brain axis—can independently modulate EEG and thus warrant explicit consideration in future RLS–EEG work. Outside the RLS field, hypothyroidism has been associated with diffuse background slowing on EEG. Thyroid autoimmunity (Hashimoto encephalopathy) produces nonspecific slowing and epileptiform activity. At the same time, hyperthyroidism has also been linked to characteristic EEG abnormalities that may persist despite treatment, indicating that even subclinical shifts in thyroid status could alter cortical rhythms or evoke responses recorded in RLS cohorts [160,161,162]. Beyond endocrinology, microbiota-driven gut–liver–brain interactions provide clear precedents: hepatic encephalopathy, in which dysbiosis and ammonia from the gut contribute to neurocognitive dysfunction [163], shows stereotyped EEG slowing and triphasic patterns, underscoring that gut-derived metabolites can reshape electrophysiology [164]. More broadly, human studies demonstrate that manipulating or differing gut states relate to EEG variation—for example, a randomized probiotic trial in preschoolers with autism reported changes in frontal beta/gamma power and coherence, and patients with disorders of gut–brain interaction such as IBS show altered auditory and visceral ERPs—implying that microbiota and visceral signaling can be bias to both resting and task-evoked EEG [165,166,167,168]. Accordingly, future EEG studies in RLS should at minimum measure TSH and free T4 (and document thyroid autoimmunity/medication), and record gut-relevant variables (e.g., recent antibiotics/probiotics, diet, and—where feasible—microbiome or permeability/inflammation markers) to enable stratified analyses or covariate adjustment.

## 5. Conclusions

Electroencephalography has evolved from a primarily descriptive tool into a mechanistic lens that now reveals RLS as a multisystem brain disorder rather than a purely peripheral motor problem. Taken together, the 15 studies reviewed show that individuals with RLS live in a state of persistent cortical hyperarousal: even during relaxed wakefulness, they display excess fast-frequency power (high-beta and low-gamma) superimposed on a mixed slow-alpha/delta shift. This abnormal “dissociated vigilance” profile, which is stronger in the evening, distinguishes RLS from healthy wakefulness and overlaps partly—but not completely—with the patterns seen in major depression and chronic insomnia. Against this tonic background, task-evoked potentials expose a clear fronto-parietal processing deficit. The late positive P300 component is systematically smaller and slower, and global-field power is reduced, indicating that the networks that allocate attention and update working memory work inefficiently. Source modeling repeatedly implicates medial prefrontal, anterior cingulate, and precuneus hubs—regions whose function depends on iron-driven dopaminergic and noradrenergic modulation—thereby linking the electrophysiology to RLS’s well-established iron–dopamine axis.

At the level of large-scale communication, phase-synchrony and graph-theoretic analyses converge on lower clustering, longer characteristic path lengths, and an overall loss of small-world efficiency. These network inefficiencies mirror clinical severity and improve—though rarely normalize—after dopaminergic therapy or iron repletion, suggesting they are both causal and modifiable. The malfunction is time-sensitive: early sensory processing, indexed by the visual N1 and flanker-interference costs, deteriorates selectively in the evening, reinforcing the idea that circadian dopamine troughs unmask latent cortical vulnerabilities and arguing for chronotherapeutic dosing of drugs, iron, and neuromodulation. Finally, EEG recorded around spontaneous or voluntary leg movements demonstrates an exaggerated post-movement β rebound and reveals that each movement is embedded in a broader sensorimotor–autonomic event: a broadband cortical surge begins about ten seconds before the kick, peaks at movement onset, then couples to a sympathetic spike that carries cardiovascular consequences far beyond sleep laboratories. These findings portray RLS as a thalamo-cortical dysrhythmia driven by brain iron deficiency, propagated through dopaminergic and GABAergic imbalance, and expressed as intertwined disturbances of attention, sensory gating, motor control, and autonomic regulation. They also supply a short list of candidate biomarkers—resting high-beta power, P300 amplitude, small-world metrics, and peri-movement β–β-heart-rate integrals—that are reproducible, sensitive to treatment, and ready for use in mechanism-based clinical trials.

## Figures and Tables

**Table 1:** Studies included in the review.

Correlations/Clinical Links	EEG/ERP Findings	Behavioral	Paradigm/Measure	Sample	Focus/Aim	Study
Parietal P300 ↓ correlated with longer illness duration; no links with PSQI/ISI/BDI	Parietal P300 amplitude ↓ in RLS across loads; load ↑ → P300 ↓, RT ↑; no group × load interaction	RLS slower RT; similar hit rate	Sternberg WM (2–4 digits); ERPs (P300) at F/C/P	13 drug-naïve severe RLS (52 y); 13 HC (52 y)	RLS vs. HC working memory (WM) via P300	[51]
P300 latency ↔ bothersomeness (VAS); no correlation with IRLS	Resting beta (26–30 Hz) ↑ frontal/central; P300 latency ↑ and amplitude ↓ esp. frontal/central (300–350 ms)	Accuracy comparable; RLS slower RT; ↑ bothersomeness (VAS); SSS similar	Resting EEG (27 ch); visual oddball; P300	17 female drug-naïve RLS (53.7 y); 13 female HC (54.6 y)	Daytime EEG/ERP abnormalities and P300 in RLS	[52]
IRLS 30.1→14.0; PSQI/ISI/BDI improved; sleep duration ↑ (ns); ESS ↔	Parietal P300 amplitude ↑ across loads; frontal P300 changes track clinical improvement	RT ↓ across loads; hit rate ↑ (88→91%); omission errors ↓; commission ↔	Modified Sternberg WM; ERPs (P300 at F/C/P)	13 drug-naïve RLS, pre/post 12–16 wks pramipexole	Effect of pramipexole on WM and P300	[53]
Supports dopaminergic frontal dysfunction	P2 amplitude ↓ frontally (Fz); source ↓ in ACC/mPFC; P3 amplitude ↓ and latency ↑; sources ↓ in ACC and precuneus; GFP ↓	Accuracy intact; faster RT in RLS (possible compensatory strategy)	Visual oddball; ERPs (P2 150–250 ms; P3 300–450 ms); LORETA	17 female RLS (53.7 y); 13 female HC (54.6 y)	Frontal involvement (P2/P3) with source localization	[54]
SWP negatively correlated with IRLS (r = −0.65)	Frontal TBA ↑ delayed and smaller; interregional TBPS ↓ (frontal-centered); graph: ↓ clustering, ↑ path length, ↓ small-world propensity	RLS slower RT; accuracy similar	Sternberg WM; EEG theta-band activity (4–6 Hz) and phase synchrony (wPLI)	12 female RLS (53.4 y); 12 female HC (49.3 y)	Frontal theta dynamics and WM retrieval	[55]
Effects independent of sleepiness; path length modestly ↔ ferritin	P300 delayed and reduced; induced gamma ↓ and delayed (frontal/central); GBPS ↓ esp. 300–500 ms; longer path length, lower clustering	RLS slower RT; ↑ bothersomeness; accuracy high	Visual oddball; ERPs (P300); induced/evoked gamma; GBPS; graph metrics	17 female RLS; 13 female HC	Gamma synchrony and network metrics during oddball	[56]
Relevance scores correlate with PSQI, ISI, IRLS	CNN accuracy ≈ 94% (AUC 0.93); relevance in L sup. frontal/parietal, temporal, insular, occipital regions	RLS slower RT; hit rate similar	Modified Sternberg WM; sLORETA features → CNN (LOOCV)	13 RLS; 13 HC → final 9 RLS after exclusions	Explainable deep learning on single-trial ERPs in WM	[57]
RLS severity negatively ↔ connectivity in sensory cortices	↑ connectivity in L primary somatosensory (BA1L); ↓ in R anterior PFC (BA10R) and R V1 (BA17R) vs. PI	No behavioral measures (resting)	Resting eyes-closed; ciPLV across bands; source-space	107 RLS (46.2 y); 17 PI (48.4 y)	QEEG connectivity: RLS vs. Primary Insomnia	[58]
Not explained by severity, sleep quality, fatigue	N1 amplitude ↓ in RLS in evening (incompatible trials); source BA18; N2/P3 ↔	RLS: larger PM interference (performance decline); HC stable	Flanker task AM (8–9) vs. PM (17–18); ERPs; sLORETA	33 RLS (65.2 y); 29 HC (64.4 y)	Circadian effects on attention (flanker) in RLS	[59]
Profiles resemble depression; PSG: not in this study	ABS delta and alpha-2 ↑; alpha-1 ↓ (parietal); REL alpha-2 ↑ (occipital/left OT/right frontal); REL beta-3 ↑ (R parietal); ABS beta-5 ↓ (R frontal); centroid shifts → ‘dissociated vigilance’	Higher depression/anxiety; poorer sleep; QoL ↓; ESS ↔	Vigilance-controlled EEG (21 ch, 3 min); spectral metrics	33 RLS (59.0 y); 33 HC (57.0 y)	Daytime EEG mapping and psychometrics in RLS	[60]
Both: ↑ PLM indices/arousals; RLS QoL ↓; PLMD ↑ sleepiness	RLS EEG: delta ↑, fast alpha ↑, slow alpha ↓; alpha centroid ↑; dom. freq ↑ but power ↓; total power ↔	RLS: worse sleep/awakening quality; AM fine motor and RT impaired; PLMD: deficits in memory/attention/motor	EEG mapping; 2-night PSG (subset); morning tests	33 RLS; 26 PLMD; 33 HC	Day/night EEG, PSG, and performance: RLS and PLMD	[61]
Suggests common cortical and sympathetic activation mechanisms	EEG power ↑ across all bands ~10 s pre-movement and post; HR ↑ from ~15 s pre-onset; strongest HR after SILM	(resting with movements)	1-h SIT; EEG spectral (δ/θ/α/β) and heart rate around PLM/ILM/SILM	53 drug-free RLS (51.9 y)	EEG/HR around leg movements during SIT	[62]
Interpreted as stronger post-movement inhibition/feedback	ERD during movement ↔; post-movement ERS markedly ↑ in RLS (C3 lower/upper beta; Cz upper beta)	(simple motor)	Right-hand button press to clicks; EEG beta ERD/ERS at C3/Cz	10 primary RLS (45.5 y); 10 HC (46.3 y)	Motor cortical ERD/ERS during simple movement	[63]
Insomnia strongly associated with beta EEG patterns	Beta activity prevalent in RLS (56.5% vs. 20% HC); mild paroxysmal slowing in 34.8% RLS	High insomnia prevalence; many on pramipexole; daytime sleepiness in 60.9%	Awake resting EEG (18 ch); activation procedures	23 RLS (52.3 y); 20 HC (47.1 y)	Awake morning EEG patterns in treated RLS	[64]
Cortical initiation for PLMW; feedback processing after PLMS	PLMS: no pre-movement ERD (subcortical/spinal) + strong PMBS; PLMW: clear pre-movement mu/beta ERD (cortical involvement)	Evening voluntary movement: RLS ERD ↑ & PMBS longer; HC ERD ↓ evening	EEG (38 ch) + EMG; PSG and SIT; voluntary ankle dorsiflexion AM vs. PM	12 idiopathic RLS (49.7 y); 10 HC (52.7 y)	Cortical role in PLMS/PLMW and voluntary movement	[65]

Abbreviations: RLS = restless legs syndrome; HC = healthy controls; WM = working memory; EEG = electroencephalography; ERP/ERPs = event-related potential(s); P2 = ERP component ~150–250 ms; P300/P3 = ERP component ~300 ms indexing attention/memory; F/C/P = frontal/central/parietal scalp regions; Fz/Pz/C3/Cz = 10–20 EEG electrode sites; RT = reaction time; HR (task) = hit rate (task accuracy); HR (physio) = heart rate; PSG = polysomnography; IRLS/K-IRLS = International Restless Legs Scale/Korean IRLS; PSQI = Pittsburgh Sleep Quality Index; ISI = Insomnia Severity Index; ESS = Epworth Sleepiness Scale; BDI-II = Beck Depression Inventory–II; VAS = Visual Analog Scale; SSS = Stanford Sleepiness Scale; ACC = anterior cingulate cortex; mPFC = medial prefrontal cortex; GFP = global field power; LORETA/sLORETA = (standardized) low-resolution electromagnetic tomography; TBA = theta-band activity; TBPS = theta-band phase synchrony; wPLI = weighted phase-lag index; GBA = gamma-band activity; GBPS = gamma-band phase synchrony; QEEG = quantitative EEG; ciPLV = corrected (imaginary) phase-locking value; BA = Brodmann area; QoL/QLI = quality of life/Quality of Life Index; PLM/PLMS/PLMW = periodic limb movements (generic)/during sleep/during wakefulness; SIT = Suggested Immobilization Test; ERD = event-related desynchronization; ERS = event-related synchronization; PMBS = post-movement beta synchronization; AM/PM = morning/evening testing; DF = dominant frequency; ABS/REL = absolute/relative (EEG power); OT = occipitotemporal (region); SMA = supplementary motor area; CNN = convolutional neural network; LOOCV = leave-one-subject-out cross-validation; LRP = layer-wise relevance propagation; PFC = prefrontal cortex; S1/V1 = primary somatosensory/primary visual cortex; ns = not significant; ch = channels (EEG); δ/θ/α/β = delta/theta/alpha/beta frequency bands. Symbols used: ↑ = increased; ↓ = decreased; ↔ = no meaningful change; → = “leads to/associated with”; ≈ = approximately; × = interaction term (e.g., group × load).

## Data Availability

No new data were created or analyzed in this study. Data sharing is not applicable to this article.
